# Kinetic Evidence for Near Irreversible Nonionic Micellar Entrapment of *N*-(2′-Methoxyphenyl)phthalimide (1) under the Typical Alkaline Reaction Conditions

**DOI:** 10.1155/2014/592691

**Published:** 2014-01-16

**Authors:** M. Niyaz Khan, Yoke-Leng Sim, Azhar Ariffin

**Affiliations:** Department of Chemistry, Faculty of Science, University of Malaya, 50603 Kuala Lumpur, Malaysia

## Abstract

The values of pseudo-first-order rate constants (*k*_obs_) for alkaline hydrolysis of **1**, obtained at 1.0 mM NaOH and within [ C_*m*_E_*n*_T] (total concentration of C_*m*_E_*n*_) range of 3.0–5.0 mM for C_12_E_23_ and 10–20 mM for C_18_E_20_, fail to obey pseudophase micellar (PM) model. The values of the fraction of near irreversible C_*m*_E_*n*_ micellar trapped **1** molecules (*F*_IT1_) vary in the range ~0–0.75 for C_12_E_23_ and ~0–0.83 for C_18_E_20_ under such conditions. The values of *F*_IT1_ become 1.0 at ≥10 mM C_12_E_23_ and 50 mM C_18_E_20_. Kinetic analysis of the observed data at ≥10 mM C_12_E_23_ shows near irreversible micellar entrapment of **1** molecules under such conditions.

## 1. Introduction

The 2-state Hartley model of micelle (i.e., hydrophilic headgroup/palisade/Stern layer, and hydrophobic core) of 1936 is still under extensive use [1]. However, relatively recent studies involving kinetic and spectrometric probes strongly favor the multistate model of micelle [2–6]. The unusual effects of pure C_12_E_23_ and mixed CTABr-C_12_E_23_ micelles on the acid-base behavior of phenyl salicylate were observed in 1999 [7]. In order to gain a better and clear understanding of this unusual finding, we started studying such effects on the rate of alkaline hydrolysis of esters and imides under variety of reaction kinetic conditions. This includes the use of reaction kinetic probe molecules of different structural features in the presence of pure C_*m*_E_*n*_ (*m*/*n* = 12/23, 16/20, 18/20, and 16/10) and mixed C_*m*_E_*n*_-CTABr micelles [8–12]. The unusual and unexpected observations of these studies are as follows. (i) The decrease of hydroxide ions from the neighborhood of micellized reaction kinetic probe molecules with the increase of *R* ( = [C_*m*_E_*n*_]_T_/[NaOH] at a constant value of [NaOH]) at a typical value of *R*_*t*_ which represents a typical value of [C_*m*_E_*n*_]_T_/[NaOH] above which *k*_obs_ versus [C_*m*_E_*n*_]_T_ data fails to obey PM model. (ii) The observed data (*k*_obs_ versus [C_*m*_E_*n*_]_T_) obey PM model at *R* ≤ *R*_*t*_. (iii) The rate of hydrolysis of reaction kinetic probe molecules almost ceased when *R* ≫ *R*_*t*_. (iv) The unusual observation of (iii) could be detected with C_12_E_23_, C_16_E_10_, and C_18_E_20_ but not with C_16_E_20_ under approximately similar conditions.

Under the typical reaction conditions of earlier studies where *R* ≫ *R*_*t*_ and the rate of reaction which could not be detected within the reaction period of more than ~24 h, the possibility of whether the cessation of the rate of reaction was due to complete or near irreversible micellar binding of one of the reactants of a bimolecular reaction has not been explored. Although the meaning of “near irreversible binding” is a subjective one, we arbitrarily consider the transition of a reversible binding to near irreversible binding if the value of *k*_obs_ changes from ~10^−4^ s^−1^ (under the reversible binding condition) to ~10^−8^ s^−1^ (under the near irreversible binding condition). The present work was initiated with an aim to find out if the cessation of the rate of reaction at ≥0.01 M C_12_E_23_ was caused by the near irreversible micellar binding of **1**. The observed results and their probable explanations are described in this paper.

## 2. Materials and Methods

### 2.1. Materials

**Figure 1:**
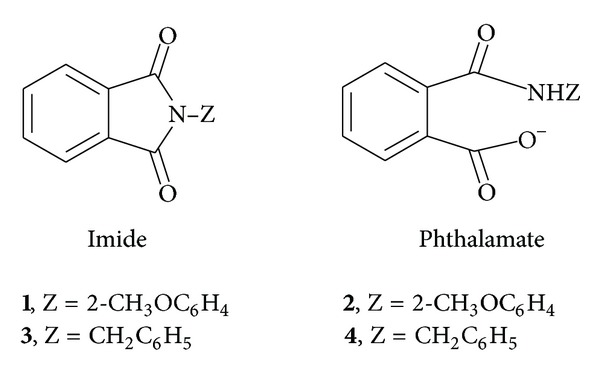
Molecular structures of compounds **1**, **2**, **3** and **4**.

Synthesis of **1** (Figure 1) has been reported earlier [14], and all the other chemicals used were commercial products of the highest available purity. Stock solutions of **1 **(5 mM and 10 mM) were prepared in acetonitrile. Throughout the text, the symbol [X]_T_ represents the total concentration of X.

### 2.2. Kinetic Measurements

The rate of nonionic micellar-mediated alkaline hydrolysis of **1** was studied spectrophotometrically at 35°C by monitoring the appearance of hydrolysis product, *N*-(2′-methoxyphenyl)phthalamate (**2**) of **1** at 290 nm as a function of reaction time, *t*. The observed data, absorbance (*A*_obs_) versus *t*, obeyed

(1) $$\begin{array}{c} A_{\text{obs}}=\delta_{\text{ap}}\left[1_{0}\right]\left[1-\exp\left(-k_{\text{obs}}t\right)\right]+A_{0}, \end{array}$$

where *k*_obs_ and *δ*_ap_ represent pseudo-first-order rate constants for alkaline hydrolysis of **1** and molar absorptivity of reaction mixture, respectively, and [**1**_0_] is the initial concentration of **1** and *A*_0_ = *A*_obs_ at *t* = 0. The details of the product characterization are described elsewhere [13]. 

## 3. Results

### 3.1. Effects of [C_12_E_23_]_T_ and [C_18_E_20_]_ T_ on Pseudo-First-Order Rate Constants (*k*_obs_) for Hydrolysis of **1** at 1.0 mM NaOH and 35°C

The rate of alkaline hydrolysis of **1** was studied within [C_12_E_23_]_T_ range of 3–50 mM, but the absorbance of the reaction mixtures within [C_12_E_23_]_T_ range of 10–50 mM remained unchanged in the reaction time (*t*) range of ~15 s–623 h. However, the observed data (*A*_obs_ versus *t*), obtained within [C_12_E_23_]_T_ range of 3–5 mM, were found to fit to (1). The least-squares calculated values of *k*_obs_, *δ*_ap_, and *A*_0_, obtained under such conditions, are shown in Table 1. Similarly, the kinetic runs for the rate of alkaline hydrolysis of** 1** were carried out within [C_18_E_20_]_T_ range of 10–50 mM. But the absorbance of the reaction mixture at 50 mM C_18_E_20_ remained unchanged within the *t* range of ~15 s–~260 h. The calculated values of *k*_obs_, *δ*_ap_, and *A*_0_ for the kinetic runs carried out within [C_18_E_20_]_T_ range of 10–20 mM are shown in Table 2.

## 4. Discussion

### 4.1. Evidence for the Near Irreversible C_12_E_23_ Micellar Binding of **1** under the Typical Reaction Conditions

It can be easily shown from the derivation of (1) that *δ*_ap_ = *δ*_2_ − *δ*_1_, where *δ*_2_ represents molar absorptivity of **2 **(Figure 1). The values of *δ*_1_ and *δ*_2_, at 290 nm, are 2480 and 5570 M^−1 ^cm^−1^ [15], respectively, in aqueous alkaline solvent containing 2% v/v CH_3_CN. The values of *δ*_1_ are independent of [C_*m*_E_*n*_]_T_ [13]. The values of *δ*_ap_ [13] reveal that the values of *δ*_2_ are also independent of [C_*m*_E_*n*_]_T_ within its range of 0.0–3.0 mM for C_16_E_20_ and C_12_E_23_ as well as 0.0–5.0 mM for C_18_E_20_. However, the values of *δ*_2_ show a nonlinear increase from 5570 to 8450 M^−1 ^cm^−1^ at 290 nm with the increase in the content of CH_3_CN from 2 to 80% v/v in mixed H_2_O-CH_3_CN solvent [15]. Thus, the decrease in *δ*_ap_ with increase in [C_*m*_E_*n*_]_T_ (Tables 1 and 2) rules out the possibility of C_*m*_E_*n*_ (*m*/*n* = 16/20, 12/23, and 18/20) micellar binding of **2** in a micellar environment of lower concentration of water compared with water concentration of bulk aqueous phase. These observations show that the effects of [C_*m*_E_*n*_]_T_ on *δ*_1_ and *δ*_2_ cannot explain the observed decrease in *δ*_ap_ with increase in [C_*m*_E_*n*_]_T_ at the typical values of [C_*m*_E_*n*_]_T_ (Tables 1 and 2). Thus, the most plausible reason for such decrease in *δ*_ap_ is due to near irreversible micellar trapping of unreacted  **1**. Under such circumstances, the observed data (*k*_obs_ versus [C_*m*_E_*n*_]_T_) listed in Tables 1 and 2 cannot be expected to obey pseudophase micellar model (PM).

It can be shown that the fraction of near irreversibly C_*m*_E_*n*_ micellar trapped **1** at *t* = *∞* (*F*_IT1_) may be given as

(2) $$\begin{array}{c} F_{\text{IT}1}=1-\left(\frac{\delta_{\text{ap}}}{{\delta_{\text{ap}}}^{\text{avg}}}\right), \end{array}$$

where *δ*_ap_ and *δ*_ap_^avg^ represent apparent molar absorptivity of the reaction mixture at *F*_IT1_ ≠ 0 and *F*_IT1_ = 0, respectively. The derivation of (2) involves the assumption that the absorbance due to medium microturbidity remains unchanged within the reaction period of *t* = 0 to *t* = *∞*. The values of *F*_IT1_ were calculated from (2) at different [C_*m*_E_*n*_]_T_ and these values are summarized in Table 1 for C_12_E_23_ and Table 2 for C_18_E_20_. It is evident from the calculated values of *F*_IT1_ that the value of [C_*m*_E_*n*_]_T_/[NaOH] ( = *R*) is nearly 3.6-fold larger for C_18_E_20_ than that for C_12_E_23_ to result in nearly same value of *F*_IT1_, while the value of *F*_IT1_ remains zero even at *R* = 170 for C_16_E_20_ [13]. The typical value of *R* ( = *R*_*t*_), at which *F*_IT1_ = 0.13, is 3.4 for C_12_E_23_. Similarly, the value of *R*_*t*_, at which *F*_IT1_ = 0.17, is 12.0 for C_18_E_20_. The values of *F*_IT1_ and *F*_IT3_ are ~0 [13] and 0.60 [11], respectively, at *R* = 170 for C_16_E_20_ micelles which reveal that the structural features of imide substrates (**1** and **3**) (Figure 1) affect the values of *F*_IT1_ at a fixed value of *R*. It is interesting and amazing to note that the difference of only 2 methylene (CH_2_) groups between C_18_E_20_ and C_16_E_20_ has so much different effects on *F*_IT1_.

If micellar entrapment of unreacted **1**, as shown by *F*_IT1_ values in Tables 1 and 2, is indeed an irreversible or near irreversible process, then the values of *A*_obs_ at *t* ≥ 10 half-lives (Reaction time *t* at ~10 half-lives is equivalent to *t*_*∞*_ because more than 99.9% reaction is progressed during the reaction period of 10 half-lives and therefore, at *t*_*∞*_, *A*_obs_ = *A*_*∞*_) should remain essentially unchanged with the increase in *t* at *t* = *t*_*∞*_ or at *t*, where *A*_obs_ = *A*_*∞*_. In order to test this conclusion, the kinetic reaction mixtures at 0.01, 0.02, 0.03, and 0.05 M C_12_E_23_ were left at 35°C for the reaction period of ~1.10 × 10^3^ h and the values of *A*_obs_, during these reaction periods, remained essentially unchanged (Table 1).

It is apparent from Tables 1 and 2 that the values of *F*_IT1_ increase nonlinearly with the increase of *R* at a typical value of *R* (=*R*_*t*_) and the values of *F*_IT1_ appear to become 1 at *R* ≥ 10 for C_12_E_23_ (Table 1) and at *R* = 50 for C_18_E_20_ (Table 2). If the reversible and near irreversible nonionic micellar binding of **1** is a function of *R*, then the change of inequality from *R* > *R*_*t*_ to *R* < *R*_*t*_, by sudden external addition of known amount of NaOH to the reaction mixture at *t* > *t*_*∞*_, must cause near irreversible bound 1_M_ molecules to become reversible bound 1_M_ molecules. Consequently, the rate of appearance of product (**2**) of this reaction mixture would follow (1) and the value of *k*_obs_ may then be compared with *k*_obs_ obtained by carrying out another kinetic run by the use of authentic sample of **1** under essentially similar experimental conditions. Such an attempt is described as follows.

To 3.0 cm^3^ of the reaction mixture containing 0.1 mM **1**, 1.0 mM NaOH, and 10 mM C_12_E_23_ (i.e., *R* = 10), 0.02 cm^3^ of 1.0 M NaOH was added at *t* = 432 h. The absorbance change of the resulting reaction mixture was quickly monitored spectrophotometrically at 290 nm as a function of reaction time (*t*). The observed data (*A*_obs_ versus *t*) were found to fit to (1) and the least-squares calculated values of kinetic parameters *k*_obs_, *δ*_ap_, and *A*_0_ are summarized in Table 3. Similar kinetic runs were carried out at different *t* (≥600 h) and [C_12_E_23_]_T_ (=0.02, 0.03, and 0.05 M) and the values of *k*_obs_, *δ*_ap_, and *A*_0_, obtained under these conditions, are also shown in Table 3.

A few kinetic runs were carried out using authentic sample of **1** freshly prepared at 35°C, 0.1 mM **1**, different values of [C_12_E_23_]_T_ (ranging from 10 to 50 mM) and [NaOH] (ranging from 4.2 to 30.0 mM). The spectrophotometrically observed data for these kinetic runs followed strictly (1) as evident from the standard deviations associated with the calculated kinetic parameters *k*_obs_, *δ*_ap_, and *A*_0_ (Table 3). The values of *k*_OH_ (=*k*_obs_/[NaOH]) are >4-fold smaller than *k*_OH_ (=36 M^−1^ s^−1^) [15] obtained under similar kinetic conditions in the absence of micelles. These results may be attributed to merely nonionic micellar inhibitory effect (the fraction of micellized **1**, i.e., 1_M_, under such conditions, is >90%, where *K*_*S*_ = 925 M^−1^ [13]).

The values of *k*_obs_, obtained from the reaction mixtures at different [C_12_E_23_]_T_ and the reaction time *t* (ranging from 432 to 1102 h) at which the value of [NaOH] was increased from 1.0 mM to ≥7.6 mM and ≤30.0 mM, are comparable with the corresponding values of *k*_obs_, obtained from authentic sample of **1 **(Table 3). These observations support the proposal of near irreversible entrapment of **1** molecules by C_12_E_23_ micelles at *R* ≫ *R*_*t*_. The observed values of *A*_obs_ at *t* ≥ 600 h as well as ≤1102 h and [C_12_E_23_]_T_ range of 10–50 mM (Table 1) reveal that the values of *F*_IT1_ must be nearly 1. But the calculated values of *F*_IT1_ at *t* ≈ 600 h, as summarized in Table 3, increase from ~0.55 to ~1.0 with the respective increase in [C_12_E_23_]_T_ from 10 to 50 mM. Similarly, the values of *F*_IT1_ at *t* range of *≈*1083–1102 h, shown in Table 3, increase from 0.51 to 0.91 with the respective increase in [C_12_E_23_]_T_ from 20 to 50 mM. These results show that, even at the highest value of [C_12_E_23_]_T_ (=50 mM) of the present study, nearly 9% hydrolysis of **1** occurred within the reaction time (*t*) of 1102 h. Thus, it is apparent that there is not any absolute/complete irreversible micellar entrapment of **1** molecules—a situation encountered with usual shielding effect of the micelles. A qualitative explanation of these observations may be described as below.

In view of the earlier reports [8, 11] on the related reaction systems, the rate of hydrolysis of **1** at 1.0 mM NaOH, 35°C, and within [C_12_E_23_]_T_ range of 0.01–0.05 M may be expected to follow an irreversible consecutive reaction path:

(3) $$\begin{array}{c} 1_{\text{M}}\overset{k_{1}}{\to }2_{\text{M}}\overset{k_{2}}{\to }\text{PA}{\text{n}}_{\text{M}}+2\text{-M}{\text{A}}_{\text{M}}, \end{array}$$

where PAn and 2-MA represent phthalic anhydride and 2-methoxyaniline, respectively, and subscript M represents micellar pseudophase. The values of *k*_2_ (at 35°C) are almost zero and 12 × 10^−4^ s^−1^ at 1.0 mM NaOH and 49 mM HCl, respectively [15]. The efficient reactivity of nonionized **2** (i.e., **2H**) towards the formation of PAn is primarily due to intramolecular carboxylic group—assisted cleavage of **2H** [15]. The respective absence and presence of the formation of PAn in the aqueous cleavage of **3** at 1.0 mM NaOH, [C_16_E_10_]_T_ ≤ 30 mM, and at [C_16_E_10_]_T_ ≥ 50 mM have been ascribed to the consequence of the effects of [C_16_E_10_]_T_ on the pH of micellar environment of nonionized **4 **(Figure 1) [11]. Spectrophotometric evidence revealed the fact that the increase in [C_12_E_23_]_T_ at *R* ≫ *R*_*t*_ with a constant value of [NaOH] caused decrease in pH of micellar environment of micellized ionized phenyl salicylate [7, 9]. In view of this study, at [C_12_E_23_]_T_ ≥ 10 mM, the pH of the micellar environment of 2_M_ dropped to a level where there was significant amount of **2H** which caused kinetically detectable occurrence of *k*_2_—step (see (3)) within [C_12_E_23_]_T_ range of 10–30 mM.

The respective values of *δ*_1_, *δ*_2_, *δ*_2**H**_, and *δ*_PAn_ (with *δ*_X_ representing molar absorptivity of X) at 290 nm are ~2420 [13], 5570–8450, 4545–7490, and 2300–2000 M^−1 ^cm^−1^ [11] within CH_3_CN content range of 2–80% v/v in mixed aqueous solvent. Close similarity of the values of *δ*_1_ and *δ*_PAn_ coupled with significantly higher values of *δ*_2_ or *δ*_2**H**_ compared with those of *δ*_1_ and *δ*_PAn_ reveal that *k*_2_ > *k*_1_. These observations explain the observed constancy of *A*_obs_ within reaction time (*t*) ranging from ~15 s to ≤1102 h at 10–50 mM C_12_E_23_ (Table 1). The rough and approximate values of *k*_1_ were obtained from the relationship: *k*_1_ = (1/*t*)ln(1/*F*_IT1_) and such calculated values of *k*_1_ at two different *t* and three [C_12_E_23_]_T_ (10, 20, and 30 mM) are shown in Table 3. It is evident from these results that the values of *k*_1_ at two different *t* and at a constant [C_12_E_23_]_T_ are comparable within the limits of experimental uncertainties. But the values of *k*_1_ decrease almost nonlinearly with the increasing values of [C_12_E_23_]_T_. Thus, the values of *k*_1_ became almost zero at 50 mM C_12_E_23_ and as a consequence only ~9% conversion of **1** to **2** could occur at *t* = 1102 h (Table 3). The values of *k*_1_ decreased from ~26 × 10^−8^ to 2.3 × 10^−8^ s^−1^ with the increase in [C_12_E_23_]_T_ from 10 to 50 mM. The values of *k*_1_ were found to decrease by ~3-fold, while the values of *k*_2_ remained unchanged with the increase of [C_16_E_10_]_T_ from 50 to 88 mM in the aqueous cleavage of **3** [11]. Although the calculated values of *k*_1_ are not very reliable because they are derived from only either two or one data point(s), these values of *k*_1_ appear to be plausible for the reason that the value of *k*_1_ at pH ~3.5, in mixed aqueous solvent containing 2% v/v CH_3_CN, is 67 × 10^−8^ s^−1^ [16]. Under such typical conditions, the value of *k*_2_ is 120 × 10^−5^ s^−1^ and it decreases from 120 × 10^−5^ to 6.6 × 10^−5^ s^−1^ with increase in CH_3_CN content from 2 to 82% v/v [15].

**Scheme 1:**
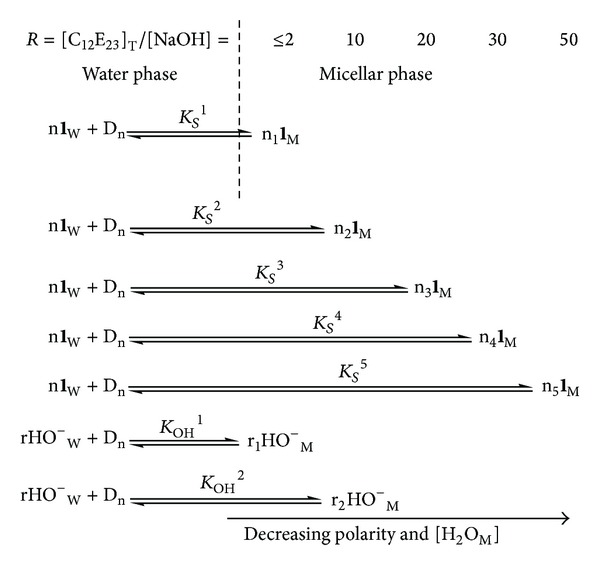


The values of *k*_obs_ and *k*_1_ show a nonlinear decrease with the increase of [C_12_E_23_]_T_ within its range of 1.0 × 10^−6^–0.05 M (Tables 1 and 3). The value of *k*_M_ (=rate constant for hydrolysis of **1** in the micellar pseudophase) remained kinetically undetectable under such conditions. The observed data failed to obey the pseudophase micellar (PM) model at >1.4 mM C_12_E_23_ because the values of micellar binding constant of **1** (*K*_*S*_) increase significantly (~10^3^-fold) with the increase in [C_12_E_23_]_T_ from 1.4 to 50 mM at 1.0 mM NaOH (Tables 1 and 3). Similar but not identical observations have been obtained in CTABr-(cetyltrimethylammonium bromide-) mediated pH-independent hydrolysis of *N*-(2-hydroxyphenyl)phthalimide [17]. The scenario exhibited by these observations supports the multicompartmental model of micelle [2, 18, 19] and it may best be represented by Scheme 1, where n_1_ **1**_M_ molecules are in equilibrium with n**1**_W_ molecules and equilibrium or micellar binding constant *K*_*S*_^1^ at *R* ≤ 2 and [NaOH] = 1.0 mM. Similarly, n_2_ **1**_M_, n_3_ **1**_M_, n_4_ **1**_M_, and n_5_ **1**_M_ molecules are in equilibrium with n**1**_W_ molecules and equilibrium constants *K*_*S*_^2^, *K*_*S*_^3^, *K*_*S*_^4^, and *K*_*S*_^5^ at respective *R* = 10, 20, 30, and 50 and a constant 1.0 mM NaOH.

## 5. Conclusions

The new and notable aspects of the present paper are the experimentally determined pseudo-first-order rate constants (*k*_1_ ≡ *k*_obs_) of the order of 10^−7^–10^−8^ s^−1^ for the hydrolysis of **1** within the *R* range of 10–50, where *R* = [C_12_E_23_]_T_/[NaOH], with a constant value of [NaOH] (= 1.0 mM). These values of *k*_1_ are >10^5^-fold smaller than *k*_obs_ at *R* ≤ 1.4, where pseudophase micellar (PM) reveals that *k*_M_ ≈ 0 and *K*_*S*_ = 925 M^−1^ [13]. The kinetic data of this paper show that the half-lives of alkaline hydrolysis of **1** at 1.0 mM NaOH and 35°C vary in the order 24 s, 6 min, 7 h, 31, 47, 122, and 349 days at *R* = 0.2, 3.4,5.0,10,20,30, and 50, respectively. Such quantitative information may be useful for designing nonionic micelles as the carrier of drug molecules containing imide functionality. These kinetic data also provide quantitative but indirect evidence for the multistate model of micelle suggested, to the best of our knowledge, in only a few reports [2–6, 18, 19].

## Figures and Tables

**Table 1:** The values of *k*_obs_, *δ*_ap_, and *A*_0_ for alkaline hydrolysis of **1** in the presence of C_12_E_23_^a^.

[C_12_E_23_]_T_ M	10^4^ *k*_obs_ s^−1^	*δ*_ap_ M^−1 ^cm^−1^	10^2^ *A*_0_	*Y*_obs_^b^	*Y*_cald_^c^	*F*_IT1_^d^
0.003	30.5 ± 1.4^e^	3012 ± 53^e^	24.8 ± 0.5^e^	11.1	3.76	0
0.0034	19.3 ± 1.0	2691 ± 48	24.8 ± 0.5	17.6	4.13	0.13
0.0038	8.31 ± 1.89	2280 ± 158	26.3 ± 1.3	40.9	4.50	0.26
0.0042	2.21 ± 0.47	1657 ± 129	25.9 ± 0.8	154	4.87	0.46
0.005	0.273 ± 0.019	770 ± 22	23.9 ± 0.1	1245	5.61	0.75
0.01	f					
0.02	g					
0.03	h					
0.05	i					

^a^[1_0_] = 0.1 mM, [NaOH] = 1.0 mM, *λ* = 290 nm, *T* = 35°C, and the aqueous reaction mixture contained 2% v/v CH_3_CN. ^b^ *Y*_obs_ = *k*_*W*_/*k*_obs_, where *k*_*W*_ = *k*_obs_ ( = 340 × 10^−4^ s^−1^ [13]) at [micelles] = 0. ^c^Calculated from the relationship: *Y*_cald_ = *ϕ* + Ψ [C_12_E_23_] with *ϕ* = 0.982 and Ψ = 925 M^−1^ [13]. ^d^The values of *F*_IT1_ were calculated from (2) with *δ*_ap_^avg^ = 3090 M^−1^ cm^−1^. ^e^Error limits are standard deviations. ^f^No change in *A*_obs_ until *t* = 600 h, where *A*_obs_ = 0.246. ^g^No change in *A*_obs_ until *t* = 1083 h, where *A*_obs_ = 0.261. ^h^No change in *A*_obs_ until *t* = 1085 h, where *A*_obs_ = 0.271. ^i^No change in *A*_obs_ until *t* = 1102 h, where *A*_obs_ = 0.286.

**Table 2:** The values of *k*_obs_, *δ*_ap_, and *A*_0_ for alkaline hydrolysis of **1** in the presence of C_18_E_20_^a^.

[C_18_E_20_]_T_ M	10^4^ *k*_obs_ s^−1^	*δ*_ap_ M^−1^ s^−1^	10^2^ *A*_0_	*Y*_obs_^b^	*Y*_cald_^c^	*F*_IT1_^d^
0.01	41.9 ± 0.3^e^	3233 ± 9^e^	27.2 ± 0.0^e^	7.92	7.89	0
0.012	15.7 ± 0.5	2637 ± 30	28.0 ± 0.1	21.1	9.27	0.17
0.014	11.6 ± 0.4	2459 ± 31	28.6 ± 0.2	28.6	10.6	0.23
0.016	7.11 ± 0.24	1239 ± 15	29.1 ± 0.1	46.7	12.0	0.61
0.018	5.89 ± 0.38	898 ± 19	29.5 ± 0.1	56.4	13.4	0.72
0.02	2.17 ± 0.22	546 ± 26	29.9 ± 0.1	153	14.8	0.83
0.05	f					

^a^[1_0_] = 0.1 mM, [NaOH] = 1.0 mM, *λ* = 290 nm, *T* = 35°C, and the aqueous reaction mixture contained 2% v/v CH_3_CN. Footnotes ^b^ and ^c^ represent respective footnotes ^b^ and ^c^ of Table 1 with replacement of [C_12_E_23_] by [C_18_E_20_] as well as *k*_*W*_ = 338 × 10^−4^ s^−1^, *ϕ* = 0.998, and Ψ = 689 M^−1^ [13]. ^d^The values of *F*_IT1_ were calculated from (2) with *δ*_ap_^avg^ = 3190 M^−1^ cm^−1^. ^e^Error limits are standard deviations. ^f^Spectrophotometrically undetectable reaction within the reaction period of ~260 h, where *A*_obs_ = 0.392.

**Table 3:** Values of *k*_obs_, *δ*_ap_, and *A*_0_ calculated from (1) for alkaline hydrolysis of **1** in the presence of C_12_E_23_ micelles^a^.

10^3^ [C_12_E_23_]_T_ M	10^3^ [NaOH] M	10^3^ *k*_obs_ s^−1^	*δ*_ap_ M^−1^ cm^−1^	10^3^ *A*_0_	*k*_OH_^b^ M^−1^ s^−1^	*F*_IT1_^c^	*R* ^d^	*t* ^e^ h	10^8^ *k*_1_^f^ s^−1^
9.9^g^	7.6	64.2 ± 0.8^h^	2088 ± 25^h^	256 ± 3^h^	8.45	0.68	1.30	432	25
9.9	7.6	66.1 ± 1.1	1691 ± 32	267 ± 3	8.70	0.55	1.30	600	27
10.0^i^	7.6	67.8 ± 0.5	3293 ± 25	260 ± 3	8.92		1.32		
19.8	11.0	44.4 ± 0.5	2127 ± 19	303 ± 2	4.04	0.70	1.82	623	16
19.8	11.0	35.0 ± 0.2	1564 ± 7	314 ± 1	3.18	0.51	1.82	1083	18
20.0^i^	11.0	51.2 ± 0.2	3344 ± 13	282 ± 1	4.65		1.82		
29.5	17.4	69.9 ± 0.9	2714 ± 45	297 ± 5	4.02	0.89	1.70	600	5.5
29.5	17.4	70.3 ± 0.3	2271 ± 92	310 ± 9	4.04	0.74	1.70	1085	7.6
30.0^i^	17.0	64.8 ± 0.3	3325 ± 16	331 ± 2	3.81		1.76		
48.5	30.0	86.3 ± 1.5	3302 ± 93	291 ± 9	2.88	1.08	1.62	622	—
48.5	30.0	88.1 ± 1.8	2789 ± 89	324 ± 9	2.94	0.91	1.62	1102	2.3
48.5^i^	30.0	78.6 ± 2.5	3365 ± 129	406 ± 13	2.62		1.62		

^a^[**1**_0_] = 0.1 mM, *λ* = 290 nm, *T* = 35°C, and the aqueous reaction mixture contained 2% v/v CH_3_CN. ^b^ *k*_OH_ = *k*_obs_ [NaOH]. ^c^ *F*_IT1_ = *δ*_ap_/*δ*_ap_^avg^ with *δ*_ap_^avg^ = 3058 M^−1^ cm^−1^. ^d^ *R* = [C_12_E_23_]_T_/[NaOH]. ^e^ *t* is reaction time (*t* ≥ *t*_*∞*_) where the kinetic reaction mixture was used for micellar entrapment experiment. ^f^Calculated from the relationship: *k*_1_ = (1/*t*)ln(1/*F*_IT1_). ^g^Value of [C_12_E_23_]_T_ after external addition of [NaOH]. ^h^Error limits are standard deviations. ^i^Reaction mixture for kinetic run was freshly prepared, where *δ*_ap_ = *δ*_ap_^avg^.
